# Treatment of stress urinary incontinence with a mobile app: factors associated with success

**DOI:** 10.1007/s00192-017-3514-1

**Published:** 2017-12-08

**Authors:** Emma Nyström, Ina Asklund, Malin Sjöström, Hans Stenlund, Eva Samuelsson

**Affiliations:** 1 Department of Public Health and Clinical Medicine, Unit of Research, Education and Development—Östersund, Umeå University, Umeå, Sweden; 2 Department of Public Health and Clinical Medicine, Umeå University, Umeå, Sweden

**Keywords:** Mobile applications, Pelvic floor muscle training, Stress urinary incontinence, Self-management

## Abstract

**Introduction and hypothesis:**

Stress urinary incontinence is common among women. First-line treatment includes pelvic floor muscle training (PFMT) and lifestyle advice, which can be provided via a mobile app. The efficacy of app-based treatment has been demonstrated in a randomized controlled trial (RCT). In this study, we aimed to analyze factors associated with successful treatment.

**Methods:**

Secondary analysis of data from the RCT. At baseline and 3-month follow-up, participants (*n* = 61) answered questions about symptoms, quality of life, background, and PFMT. Success was defined as rating the condition as much or very much better according to the validated Patient Global Impression of Improvement questionnaire. Factors possibly associated with success were analyzed with univariate logistic regression; if *p* < 0.20, the factor was entered into a multivariate model that was adjusted for age. Variables were then removed stepwise.

**Results:**

At follow-up, 34 out of 61 (56%) of participants stated that their condition was much or very much better. Three factors were significantly associated with success: higher expectations for treatment (odds ratio [OR] 11.38, 95% confidence interval [CI] 2.02–64.19), weight control (OR 0.44 per kg gained, 95% CI 0.25–0.79), and self-rated improvement of pelvic floor muscle strength (OR 35.54, 95% CI 4.96–254.61). Together, these factors accounted for 61.4% (Nagelkerke R^2^) of the variability in success.

**Conclusion:**

These results indicate that app-based treatment effects are better in women who are interested in and have high expectations of such treatment. Also, the findings underline the importance of strengthening the pelvic floor muscles and offering lifestyle advice.

## Introduction

Urinary incontinence is defined as any involuntary loss of urine [1]. It is common among women, with a reported prevalence varying between 13 and 71% in different cohorts [2], and its impact on quality of life at the population level is high compared with many other conditions [3]. The most common type is stress urinary incontinence (SUI), defined as leakage upon exertion [1]. Pelvic floor muscle training (PFMT) is the first-line treatment, along with recommendations for lifestyle changes [4, 5]. There is no gold standard for how to provide treatment with PFMT; however, the current recommendation is that the most intensive treatment available should be offered [4]. A Cochrane review from 2014 concludes that among women who received PFMT, 55% reported cure or improvement compared with 3.2% of women who received no treatment [6].

Nevertheless, many women do not seek care. A recent Polish study showed that women suffered from SUI for 17 years on average before seeking medical help [7]. First-line treatment for SUI can be offered without face-to-face contact, for example, via the Internet or smartphone [8, 9]. This form of self-management could increase access to care and enable women to seek it who otherwise would not do so.

The mobile app Tät® for women with SUI with a focus on PFMT was developed within the eContinence project with the aim of improving availability and adherence to this efficient and, in theory, simple treatment. In a randomized controlled trial (RCT), treatment delivered via the app showed a clinically relevant effect on symptoms and quality of life, with significant reductions in incontinence episode frequency (IEF) and pad use. These effects were significantly greater than in the control group (postponed treatment), and patient satisfaction was generally high with the app-based intervention [8].

To our knowledge, this is the first mobile app with PFMT for management of urinary incontinence to be scientifically evaluated, and the advantages and disadvantages of app-based treatment are largely unknown. Two important and unanswered questions are whether or not app-based treatment should target certain groups of women, and if there are ways of adjusting the treatment to allow participants to benefit more. Thus, this study aimed to find factors associated with a successful outcome among women who used the mobile app for treatment of SUI.

## Materials and methods

This study is based on data collected in and alongside an RCT conducted in Sweden, in which 123 adult women with SUI at least once weekly were randomized either to treatment via a mobile app or to postponed treatment (control group). The treatment app Tät® focuses on PFMT, but includes information about SUI, lifestyle advice, reminders to do the PFMT, and statistics functions. The most important exclusion criteria were pregnancy, former urinary incontinence surgery, malignancy in the lower abdomen, visible blood in the urine, difficulties passing urine, and neurological disease affecting the lower abdomen or legs. Effects were evaluated at follow-up after 3 months of treatment. Details and results of the RCT analyses have been published elsewhere [9]. After the study, the treatment app was made available in Swedish and English free of charge.

In the current study, only those women who received the app-based treatment were included (*n* = 62). All variables were self-reported, and we never met the women face-to-face or controlled the stated information in any other way. The participants answered online questionnaires for baseline and follow-up, and IEF was measured with 2-day leakage diaries that were sent to us by mail. Validated patient-reported outcome measures were used for symptom severity (ICIQ-UI SF, International Consultation on Incontinence Modular Questionnaire—Urinary Incontinence Short Form) [10], condition-specific health-related quality of life (ICIQ-LUTSqol, International Consultation on Incontinence Modular Questionnaire—Lower Urinary Tract Symptoms Quality of Life) [11, 12], and Patient Global Impression of Improvement (PGI-I) [13].

### Definition of success

Success was defined in accordance with the PGI-I, a validated single-item questionnaire asking the participants to rate their condition now compared with before treatment [13]. The seven possible answers ranged from “very much better” to “very much worse.” In these analyses, the participants who rated themselves to be much or very much better were considered to have had a successful treatment at 3-month follow-up. This definition was chosen to ensure a comprehensive and patient-centered definition of success, and also because it is a validated questionnaire [13], and the definition has been used in previous studies [14, 15].

### Baseline factors analyzed for association with success

At inclusion, the participants answered the questionnaires for symptoms (ICIQ-UI SF) and impact on quality of life (ICIQ-LUTSqol) and filled out the leakage diaries (IEF). These were analyzed as continuous variables. The validated incontinence severity index [16] was used to categorize the total ICIQ-UI SF score into severity categories [17]: slight (1–5), moderate (6–12), severe (13–18), or very severe (19–21). Using this categorization, we also analyzed ICIQ-UI SF as a categorical variable.

In addition, the participants provided information about age, educational level, their use of the Internet and smartphones, and previous medical history, including urinary incontinence and gynecological history. With the exception of age, these factors were analyzed as categorical variables.

Additionally, the participants responded to questions about different lifestyle factors: physical activity, smoking, coffee and tea consumption. These were analyzed as categorical variables. For the analysis, we calculated a body mass index (BMI) from the reported height and weight (kg/m^2^). BMI and weight were analyzed as continuous variables.

**Fig. 1:**
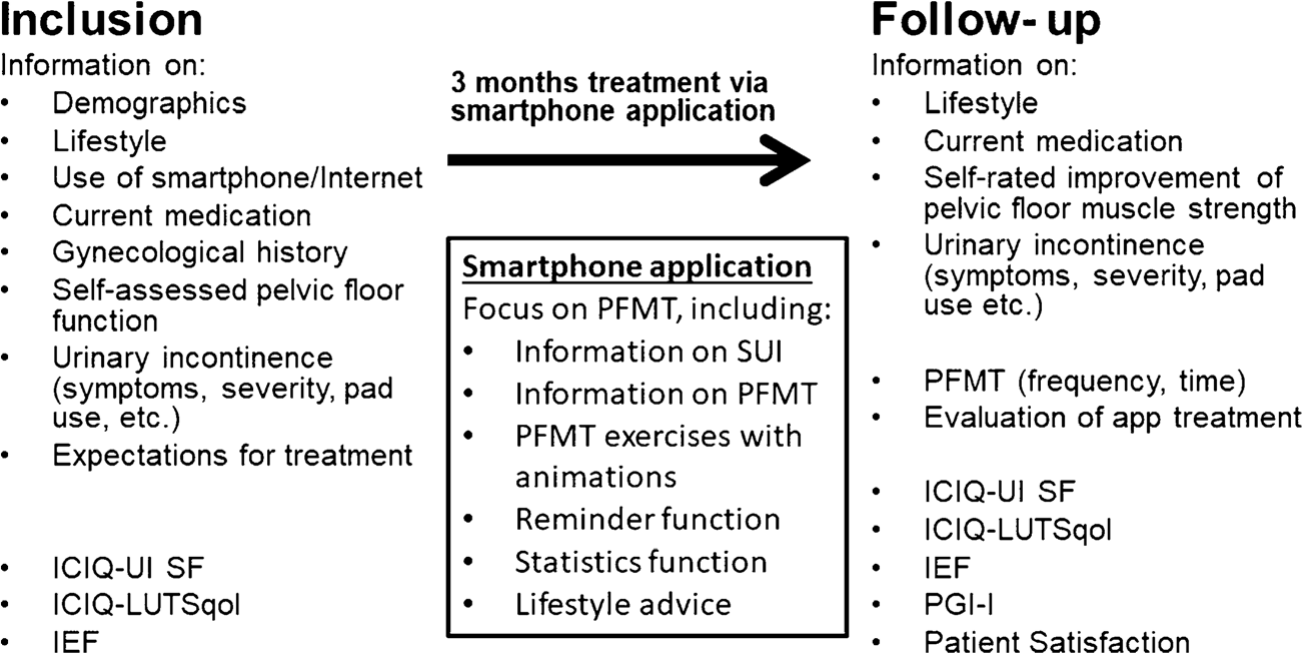
Data collected at inclusion and follow-up and features of the mobile app for treatment of stress urinary incontinence in women. *ICIQ-UI SF* International Consultation on Incontinence Modular Questionnaire—Urinary Incontinence Short Form, *ICIQ-LUTSqol* International Consultation on Incontinence Modular Questionnaire—Lower Urinary Tract Symptoms Quality of Life, *IEF* incontinence episode frequency, *PFMT* pelvic floor muscle training, *SUI* stress urinary incontinence, *PGI-I* Patient Global Impression on Improvement

Respondents also expressed their expectations for treatment by rating how they anticipated their condition would change, using a 5-point scale from “will remain unchanged” to “to be completely free of leakage.” Expectations for treatment were analyzed as a categorical variable (Fig. 1).

### Follow-up factors analyzed for association with success

At follow-up, the participants completed the lifestyle questions again and the change from baseline was analyzed. For example, we calculated the difference in weight between follow-up and baseline (kg_follow-up_ – kg_baseline_) and analyzed it as a continuous variable.

Information was also collected on the use of statistics and other functions in the app. The frequency of PFMT was measured both as an estimation of how often they had been training during the last 4 weeks and as the total number of exercises registered by the statistics function of the app. The former was analyzed as a categorical variable and the latter as a continuous variable. Further, the PFMT contractions per day were calculated from the statistics function by multiplying the exercises performed by the number of contractions per exercise; values from all levels were summarized and then divided by 90 to yield the mean contractions performed per day. The number of contractions per day was divided into four categories (<15, 15–29, 30–44, ≥45), as in a previous study by Borello-France et al. [18], and was also analyzed as a categorical variable.

In addition, the participants also subjectively rated their improvement in pelvic floor muscle strength from “much worse” to “much better” by answering the question, “How is your tightening capacity [of the pelvic floor] now compared to before the study started?” (Fig. 1). This self-rated improvement in pelvic floor muscle strength was analyzed as a categorical variable.

### Statistics

All variables from baseline and follow-up that could theoretically influence treatment success were first analyzed using univariate logistic regression to find a significant association. If data were missing, the participant was included in the analysis with missing values, i.e. no values were imputed. Symptom and quality-of-life scores (ICIQ-UI SF and ICIQ-LUTSqol), IEF, BMI, weight change, and mean PFMT frequency over the treatment period were analyzed as continuous variables both in univariate and multivariate analyses. The ICIQ-UI SF and PFMT contractions per day were also analyzed as categorical variables. All other information was collected as answers to multiple-choice questions and analyzed as categorical variables, in some cases collapsed into two or three categories. Collapsing was done when it was logically possible to minimize the risk of failing to detect significant correlations owing to a small sample size.

If the association in the univariate analysis was significant or close to significance (*p* < 0.20), the variable was entered into the multivariate model. The cut-off *p* < 0.20 was set to allow for no more than eight variables in the final model. Age is a known risk factor for urinary incontinence and increased severity [19]; thus, this variable was adjusted for throughout the analysis.

In the multivariate model, the variables were then removed one at a time according to significance level until only age and variables significantly associated (*p* < 0.05) with success remained. All analyses were performed using the statistical software SPSS version 22.0.

## Results

In total, 62 women were randomized to treatment with the mobile app; only 1 was completely lost to follow-up, and the remaining participants were included in this analysis (*n* = 61). One woman did not complete the ICIQ-UI SF, and 9 women did not use the statistics function in the mobile app; therefore, they have missing values in the analyses based on these answers. Aside from these instances, there were no missing answers to the variables included in the regression analyses.

The mean age of the participants was 45 years (range 27–72 years) and most (57.4%) had not previously sought medical care for their incontinence. Almost all had a high educational level (90.2% had attended university) and had given birth to at least one child (91.8%). Most participants had moderate or severe urinary incontinence symptoms; the mean ICIQ-UI SF score was 11.1, and the median IEF was three leakage episodes per day. For other baseline data, see Table 1.

**Table 1:** Baseline characteristics of women (*n* = 61) who received treatment for SUI via a mobile app with a focus on pelvic floor muscle training

Characteristic	Data
Demographics	
Age in years, mean (SD)	44.7 (9.7)
Education, *n* (%)	
No higher education	6 (9.8)
University studies <3 years	4 (6.6)
University studies ≥3 years	51 (83.6)
Lifestyle	
Daily smoking, *n* (%)	2 (3.3)
Body mass index kg/m^2^, mean (SD)	24.0 (4.1)
Physical activity in leisure time, *n* (%)	
Sedentary leisure time	1 (1.6)
Moderate activity but without perspiration (e.g., walking)	17 (27.9)
Regular exercise 1–2 times/week (e.g., jogging, swimming)	18 (29.5)
Regular exercise ≥3 times/week	25 (41.0)
Smartphone use	
Smartphone type, *n* (%)	
iOS users	40 (65.6)
Android users	21 (34.4)
Use of smartphone to find health information, *n* (%)	
Never	9 (14.8)
Occasionally, but not weekly	29 (47.5)
Weekly	23 (37.7)
Gynecology	
Parity, *n* (%)	
Nulliparous	5 (8.2)
Uniparous	11 (18.0)
Multiparous	45 (73.8)
Postmenopausal, *n* (%)	10 (16.4)
Incontinence	
Previously sought medical care for incontinence, *n* (%)	26 (42.6)
ICIQ-UI SF score, mean (SD)	11.1 (3.0)
Incontinence episode frequency per week, median (range)	21.0 (0.0–73.5)
Daily pad use, *n* (%)	13 (21.3)
Expectations about treatment, *n* (%)	
To be completely free of leakage	13 (21.3)
To be very much improved	27 (44.3)
To be much improved	21 (34.4)
To be a little improved	0 (0)
To experience no change	0 (0)

*SUI* stress urinary incontinence, *PFMT* pelvic floor muscle training, *SD* standard deviation, *ICIQ-UI SF* International Consultation on Incontinence Modular Questionnaire—Urinary Incontinence Short Form

**Fig. 2:**
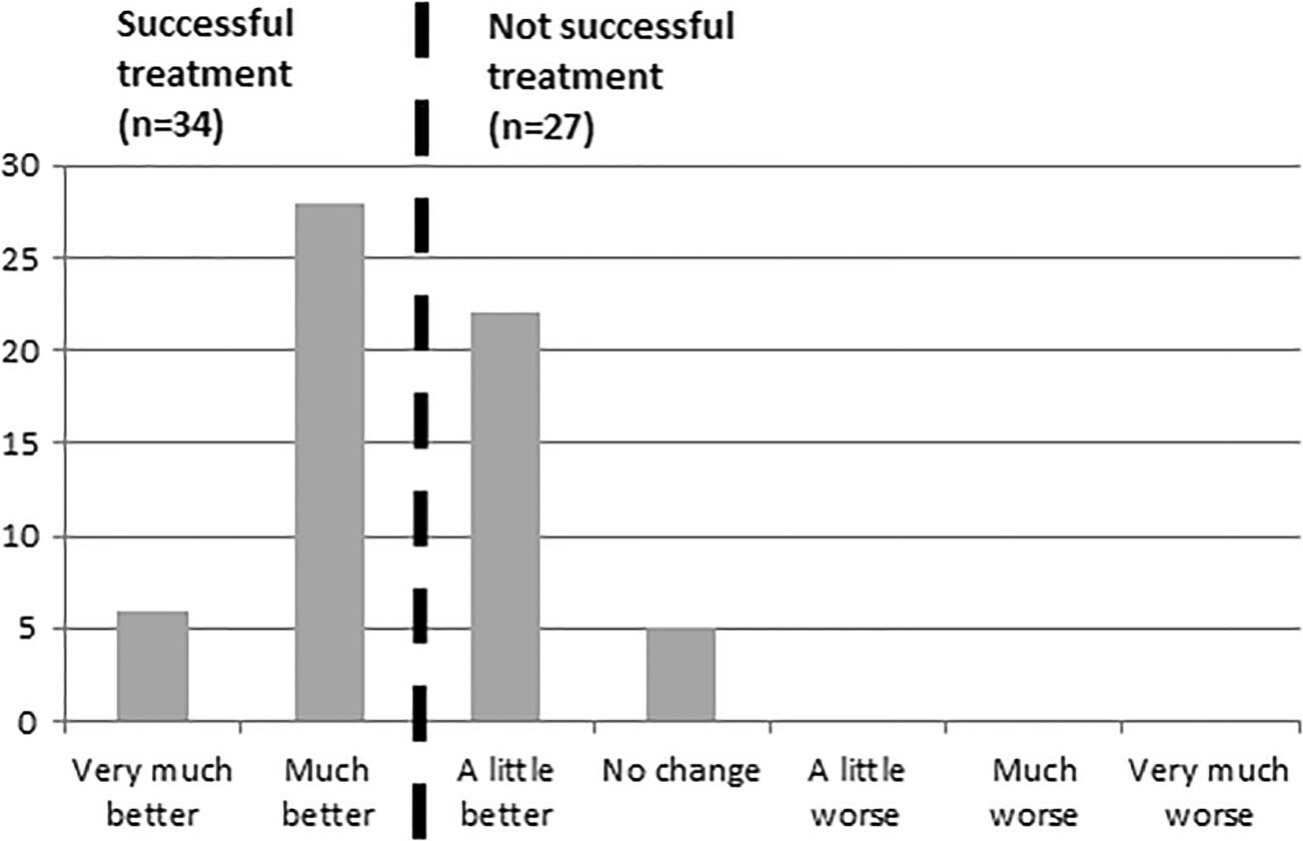
Patient Global Impression of Improvement (PGI-I) at 3-month follow-up and definition of successful outcome after treatment for stress urinary incontinence

At follow-up, 55.7% of the women perceived themselves to be “much” or “very much better” and were considered to have had a successful treatment outcome (Fig. 2). In univariate analyses, four baseline factors were significantly or borderline significantly (*p* < 0.20) associated with success, according to the PGI-I: expectations for treatment, daily tea consumption, pad use, and level of physical activity (Table 2). A further three factors at follow-up were identified: weight change during the treatment period, self-rated improvement of pelvic floor muscle strength after treatment, and categorized PFMT contractions per day (Table 3). Factors from both baseline and follow-up were included in the multivariate analyses.

**Table 2:** Univariate analysis of baseline factors

Factors possibly associated with success	Successful^a^	Not successful^a^	p*	Crude OR (95% CI)
Baseline demographics				
Age, years	43.79 (SD 9.68)	45.93 (SD 9.85)	0.395	0.98 (0.93–1.03)
Education				
No higher education or university studies <3 years	6 (17.6%)	4 (14.8%)	Reference	1.0
University studies ≥3 years	28 (82.4%)	23 (85.2%)	0.767	0.81 (0.20–3.23)
Lifestyle				
Tea consumption				
≥ 3 cups/day	2 (5.9%)	8 (29.6%)	Reference	1.0
< 3 cups/day	32 (94.1%)	19 (70.4%)	*0.023*	*6.74 (1.29–35.09)*
Coffee consumption				
≥ 3 cups/day	17 (50.0%)	15 (55.6%)	Reference	1.0
< 3 cups/day	17 (50.0%)	12 (44.4%)	0.666	1.25 (0.45–3.45)
Body mass index	23.99 (SD 3.65)	24.03 (SD 4.73)	0.974	1.00 (0.88–1.13)
Physical activity				
Regular exercise ≥3 times/week	11 (32.4%)	14 (51.9%)	Reference	1.0
Sedentary lifestyle or modest exercise <3 times/week	23 (67.6%)	13 (48.1%)	*0.127*	*2.25 (0.79–6.38)*
Baseline incontinence characteristics				
ICIQ-UI SF total score at baseline	11.18 (SD 3.15)	11.11 (SD 2.89)	0.927	1.01 (0.85–1.20)
Pad use				
Daily	5 (14.7%)	8 (29.6%)	Reference	1.0
Weekly	17 (50.0%)	13 (48.1%)	0.277	2.09 (0.55–7.91)
More seldom	12 (35.3%)	6 (22.2%)	*0.125*	*3.20 (0.72–14.15)*
Expectations of treatment				
To be much improved	7 (20.6%)	14 (51.9%)	Reference	1.0
To be very much improved/completely free of leakage	27 (79.4%)	13 (48.1%)	*0.013*	*4.15 (1.35–12.77)*

*CI* Confidence Interval, *ICIQ-UI SF* International Consultation on Incontinence Modular Questionnaire – Urinary Incontinence Short Form, *OR* odds ratio, *SD* standard deviation

*Based on univariate logistic regression. Significant (*p* < 0.05) and borderline significant (*p* < 0.20) associations are written in *italics*

^a^Means (SD) are presented if the variable has been analyzed as a continuous variable and numbers (%) if categorized or dichotomized

**Table 3:** Univariate analysis of factors at the 3-month follow-up

Factors possibly associated with success	Successful^b^	Not successful^b^	p*	Crude OR (95% CI)*
Weight change (per kg gained)	−0.29 (SD 1.70)	1.22 (SD 2.72)	*0.020*	*0.69 (0.50–0.94)*
Exercise frequency, times/week^a^	11.47 (4.89)^a^	11.82 (8.06)^a^	0.844	0.99 (0.91–1.08)
Exercise amount, contractions/day^a^				
< 15	11 (32.4%)	12 (44.4%)	Reference	1.0
15–29	14 (41.2%)	6 (22.2%)	*0.146*	*2.55 (0.72–8.96)*
30–44	4 (11.8%)	4 (14.8%)	0.916	1.09 (0.22–5.45)
≥ 45	0 (0%)	1 (3.7%)	1.000	0.00
PFMT frequency last treatment month				
Never/sporadic	4 (11.8%)	6 (22.2%)	Reference	1.0
Weekly	14 (41.2%)	12 (44.4%)	0.459	1.75 (0.40–7.70)
Daily	16 (47.1%)	9 (33.3%)	0.202	2.67 (0.59–12.02)
Self-rated improvement in pelvic floor muscle strength				
Unchanged/a little better	10 (29.4%)	22 (81.5%)	Reference	1.0
Much better	24 (70.6%)	5 (18.5%)	*<0.001*	*10.56 (3.12–35.75)*

*CI* Confidence Interval, *OR* odds ratio, *PFMT* pelvic floor muscle training, *SD* standard deviation

*Based on the univariate logistic regression. Significant (*p* < 0.05) and borderline significant (*p* < 0.20) associations are written in *italics*

^a^Based on the statistics function (*n* = 37)

^b^Means (SD) are presented if the variable has been analyzed as a continuous variable and numbers (%) if categorized or dichotomized

The seven variables in the multivariate analysis were removed stepwise until three with a significant association remained. The remaining three were expectations for treatment effect (odds ratio [OR] 11.38, 95% confidence interval [CI] 2.02–64.19), weight control (OR 0.44 per kg gained, 95% CI 0.24–0.79), and self-rated improvement of pelvic floor muscle strength (OR 35.54, 95% CI 4.96–254.61; Table 4). Together, these variables explained 61.4% (Nagelkerke R square) of the variability in success.

**Table 4:** Adjusted OR for predictors of success

Significant factors in the multivariate model (reference category)	p*	Adjusted OR (95% CI)*
Expectations of treatment (to be much improved)		
To be very much improved/completely free of leakage	0.006	11.38 (2.02–64.19)
Weight change (per kg gained)	0.006	0.44 (0.24–0.79)
Self-rated pelvic floor muscle strength (unchanged/a little better)		
Much better	<0.001	35.54 (4.96–254.61)

*CI* confidence interval, *OR* odds ratio

*Results from the multivariate logistic regression model: *p* < 0.05 was considered significant

## Discussion

In this study, expectations of treatment, weight change during treatment, and self-rated improvement of pelvic floor muscle strength were significantly associated with a successful outcome of mobile app-based SUI treatment. Higher expectations for treatment and the self-rated improvement of pelvic floor muscle strength meant a higher likelihood of a successful outcome, whereas weight gain meant a lower likelihood of success. Together, these three factors accounted for more than half of the variability in success.

### Strengths and limitations

The strengths of this study were the high follow-up rates, with only one participant lost to follow-up, and the use of a validated outcome measure as the definition of success. Other strengths were the prospective design and the clinical relevance, as all participating women actively sought this sort of care. Most importantly, however, this study evaluated a completely new way of delivering treatment, which has the potential to reach a much larger population than other treatment formats.

The major limitation is the small sample size and the large CIs stemming from this, which makes it difficult to determine the exact impact of the factors. Factors with weaker associations with success may not have been identified in our analysis. Furthermore, variability was lacking for some of the variables, such as educational level, physical activity, and smoking, and all data were self-reported. However, the factors we found to have a definitive association will most likely also be associated in larger samples. Another limitation was the study design; this investigation was a secondary analysis of data originally collected for the RCT. Furthermore, the results may not be completely generalizable to other levels of care or clinical settings because the participants in our study were highly educated and motivated for this type of treatment based on self-management.

### Our findings and their relation to previous findings

The first of our findings, the association between high expectations of treatment and success, has not been evaluated in previous studies on urinary incontinence management. The effect could be an expression of the perception of self-efficacy, intention to adhere to treatment, and positive attitude toward treatment. These factors have previously been identified as determinants of adherence [20]. However, in our small sample, we found no association between success and higher frequency of PFMT and find it unlikely that this is the full explanation. Other studies have found that factors associated with treatment failure were poor outcome of previous physiotherapy for urinary incontinence, more severe incontinence symptoms, long symptom duration, and comorbidity with other mental or physical conditions [21, 22]. All are factors that could lower not only expectations, but also motivation and the ability to conduct training. Another factor that could influence motivation and ability is education. Educational level had no association with success in our study, but has been linked to treatment outcome in other studies, although not for all outcome measures and with no association with PGI-I [14, 21]. Another possible explanation is self-efficacy. Self-assessed ability to carry out PFMT predicts long-term success [15], but has not been evaluated in other studies of predictors. The benefit of high educational level found in other studies [14] and the influence of expectations for treatment found in our study could both be surrogate measures of self-efficacy. More research is needed, however, to further evaluate the relationship between expectations for treatment, patient treatment preferences, and self-efficacy to adhere to treatment, and if patient empowerment could increase both expectations and treatment results.

Second, in our study, success and weight control were associated even in normal-weight women, which is in line with current recommendations suggesting weight loss in overweight women [4], but is not well studied during active treatment for urinary incontinence. We have found one other study in which high BMI at baseline predicted a poorer prognosis [21]. Our results further strengthen these recommendations and may even support an extension to also recommend that women with SUI do not gain weight during treatment.

The third factor associated with success was the self-rated improvement of pelvic floor muscle strength. This is in line with the correlation between response to the PFMT and increase in muscle strength measured with a vaginal balloon shown in a study by Bø and Hilde [23]. Similar results were found in a study by Hung et al., in which pelvic floor muscle strength was measured with digital palpation [24]. In line with these results, poor contraction at baseline predicted success in one other study of PFMT and bladder training [25]. From other reports, we know that short-term results predict long-term outcomes [15, 26]. The participants in our study received the app-based treatment without any physical examination or face-to-face care. In this setting, the association with success could indicate that those who did not find their pelvic floor muscle strength improved after 3 months would benefit from physical examination or personalized instruction to find the correct pelvic floor muscles and improve the chances of long-term success.

In several studies, less severe SUI symptoms, treatment naivety, or previous good treatment results predicted success [14, 15, 21, 22, 27]. These factors are most likely to co-vary with the ability to train pelvic floor muscle strength and attitudes to treatment, as they could indicate lower rates of birth injuries to the pelvic floor, for example, or no previous treatment failures that could potentially lower the adherence to the next treatment. However, in our small sample, the severity of incontinence and treatment naivety were not significantly associated with success.

Other factors predicting success found in previous studies, such as menopausal status [14], age, and physical activity [15], were also analyzed in our study, but could not be confirmed. Whether or not these factors are influential to a lesser extent is for larger studies to assess.

## Conclusion

From our results, we conclude that self-management of SUI via a mobile app can favorably be offered to women who seek such treatment and have high expectations of it, because they have higher success rate. Our results further show that weight control is beneficial, even for normal-weight women and that self-rated improvement of pelvic floor muscle strength is important for treatment success.
